# Evaluation of the Diagnostic Accuracy of GPT-4 in Five Thousand Rare Disease Cases

**DOI:** 10.1101/2024.07.22.24310816

**Published:** 2024-07-22

**Authors:** Justin T Reese, Leonardo Chimirri, Daniel Danis, J Harry Caufield, Kyran Wissink, Elena Casiraghi, Giorgio Valentini, Melissa A. Haendel, Christopher J Mungall, Peter N Robinson

**Affiliations:** 1. Division of Environmental Genomics and Systems Biology, Lawrence Berkeley National Laboratory, Berkeley, CA, 94720, USA.; 2. Berlin Institute of Health (BIH), Charité Universitätsmedizin Berlin, 10117 Berlin, Germany.; 3. The Jackson Laboratory for Genomic Medicine, Farmington CT, 06032, USA.; 4. Utrecht University, Heidelberglaan 8, 3584 CS Utrecht, Netherlands; 5. AnacletoLab, Dipartimento di Informatica, Università degli Studi di Milano, Milano, Italy.; 6. ELLIS-European Laboratory for Learning and Intelligent Systems.; 7. University of North Carolina at Chapel Hill, Chapel Hill, NC, USA

## Abstract

Large language models (LLM) have shown great promise in supporting differential diagnosis, but 23 available published studies on the diagnostic accuracy evaluated small cohorts (number of cases, 30–422, mean 104) and have evaluated LLM responses subjectively by manual curation (23/23 studies). The performance of LLMs for rare disease diagnosis has not been evaluated systematically. Here, we perform a rigorous and large-scale analysis of the performance of a GPT-4 in prioritizing candidate diagnoses, using the largest-ever cohort of rare disease patients. Our computational study used 5267 computational case reports from previously published data. Each case was formatted as a Global Alliance for Genomics and Health (GA4GH) phenopacket, in which clinical anomalies were represented as Human Phenotype Ontology (HPO) terms. We developed software to generate prompts from each phenopacket. Prompts were sent to Generative Pre-trained Transformer 4 (GPT-4), and the rank of the correct diagnosis, if present in the response, was recorded.

The mean reciprocal rank of the correct diagnosis was 0.24 (with the reciprocal of the MRR corresponding to a rank of 4.2), and the correct diagnosis was placed in rank 1 in 19.2% of the cases, in the first 3 ranks in 28.6%, and in the first 10 ranks in 32.5%. Our study is the largest to be reported to date and provides a realistic estimate of the performance of GPT-4 in rare disease medicine.

## Introduction

Large language models (LLMs) are general-purpose artificial intelligence models that can be applied to numerous tasks across diverse domains. LLMs display excellent performance in many clinical tasks.^1,2^ For differential diagnostic support, for example, a narrative text describing a patient’s features is presented to an LLM and the LLM is requested to return a ranked list of potential diagnoses.^3^

Numerous publications have addressed the accuracy of LLMs in differential diagnostics. All 23 publications we identified used human curators, usually physicians, to compare the response of LLMs (and most frequently, OpenAI’s GPT models) to the correct diagnosis recorded in the original source. This step, although conceptually simple, requires that the curator have specialized medical knowledge of the disease in question. For instance, in case Case 2–2021 of the New England Journal of Medicine Case Record series that has been used by multiple groups to assess LLM performance,^3–6^ the final diagnosis is given as “pregnancy-associated myocardial infarction, probably due to spontaneous coronary-artery dissection”.^7^ In our analysis of this case,^8^ GPT-4 returned answers including peripartum cardiomyopathy and heart failure secondary to severe pre-eclampsia, which are not correct. These answers could be assessed as “the suggestions included something closely related that might have been helpful” using the evaluation system of one of the published assessments of GPT-4 and the NEJM case reports. As a second example, in case 16–2021, the final diagnosis was “*Staphylococcus aureus* bacteremia and infection of a vascular graft“.^9^ In our study, the diagnosis at rank 4, “infective endocarditis affecting the aortic valve and causing referred abdominal pain” was reminiscent of the correct diagnosis. In this study, the two scorers agreed on only 66% of scores in 80 NEJM cases.^3^ Therefore, the practical utility of LLM analysis may be limited by the ability of users to interpret the responses. For this reason, we concluded that a computational approach to evaluate the responses of GPT-4 by assigning them to specific disease entities (Mondo ontology terms) would provide a more realistic assessment of the utility of GPT-4 for most physicians.

The 23 published studies analyzed cohorts of between 30 and 422 cases (mean 104), often from published vignettes intended for medical education, such as the *Case Studies* of New England Journal of Medicine,^3–6^ the *Diagnosis Please* quizzes from the journal Radiology,^10^ and JAMA Ophthalmology *Clinical Challenges*^11^. The level of detail available in such reports may exceed that typically available in many clinical settings. None of the published studies specifically focused on rare disease, an area of great need because affected individuals often go years before receiving a diagnosis. Over 10,000 rare diseases have been identified to date, collectively affecting between 3.5% and 8% of the population, yet many patients experience a long diagnostic odyssey of 5–7 years.^12,13^

Here, we assess the diagnostic ability of LLMs in a large set of cases of Mendelian or chromosomal diseases, leveraging a collection of computational case reports recorded using the Global Alliance for Genomics and Health (GA4GH) Phenopacket Schema.^14^ Human Phenotype Ontology (HPO) terms represented the signs, symptoms, abnormal imaging findings and laboratory test results observed or excluded in a patient. An LLM prompt is generated from this information that by design does not contain personal identifiers, genetic data, biometric data, or insurance information. We used this approach to process more than 5000 clinical narratives describing 378 rare diseases, the largest reported number of cases and diseases to be analyzed with LLMs to date.

## Methods

### Study design and data

We evaluated the performance of GPT-4 in differential diagnosis using 5267 computational case reports formatted as GA4GH phenopackets taken from the phenopacket-store repository (version 0.14).^15^ The case reports describe 378 Mendelian and chromosomal diseases associated with 336 genes. Each phenopacket contains information derived from published case or cohort reports from a total of 726 different publications. The diagnosis indicated in the original publication was recorded but not used in generating the prompt. A total of 2975 distinct HPO terms were used, with an average of 16 HPO terms per case.

### Retrieval of relevant literature

A search was conducted in PubMed to retrieve articles that describe the use of large language models for differential diagnostics. The search string was:


(“GPT”[Title/Abstract] OR
“LLM”[Title/Abstract] OR
“Large language model”[Title/Abstract])
AND
(“differential diagnostics”[Title/Abstract] OR
“differential diagnosis”[Title/Abstract])


This search was performed on July 17, 2024, and returned 80 articles. This list was further refined by selecting only articles that described the application of one or more LLMs to perform differential diagnostic analysis on a cohort of clinical cases. Further, for better comparability, we only retained publications in which the rate of placing the correct diagnosis in rank 1 was reported. For instance, we omitted one publication because only the rate of the correct diagnosis in the top three candidates was reported.^16^ The reference lists of chosen publications were scanned to identify additional articles. A full list of included articles is provided in Supplemental Table 1.

### Computational generation of prompts for GPT-4

**Figure 1.**
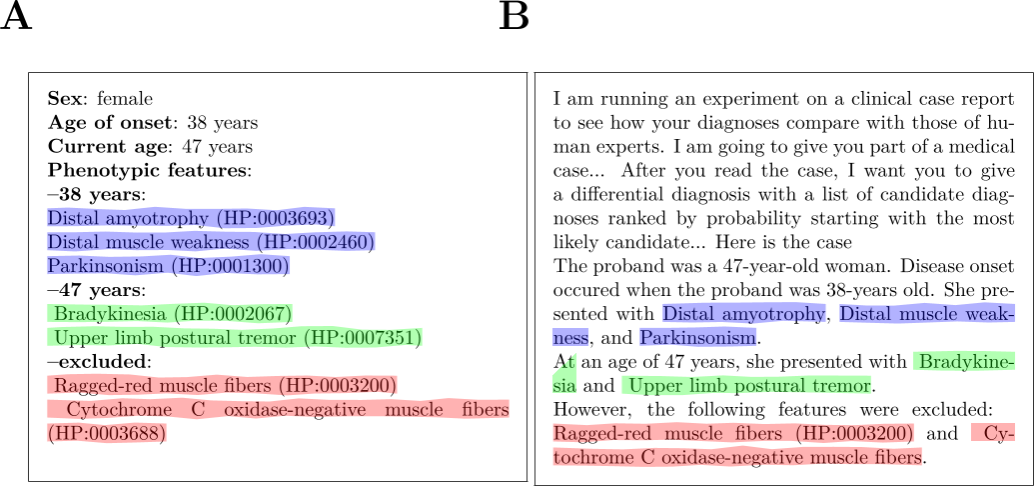
Generating prompts from structured data. A) Structured representation of a clinical case report with basic demographic information together with signs, symptoms and findings encoded as Human Phenotype Ontology (HPO) terms. Each case report is represented in JSON according to the GA4GH Phenopacket Schema (a simplified version is shown here). B) A prompt suitable for GPT-4 is generated from the phenopacket in A using phenopacket2prompt.

We constructed software, *phenopacket2prompt*, to convert case data in GA4GH Phenopacket format to prompts suitable for use with LLMs such as GPT-4 to generate differential diagnoses. It does this by parsing phenopackets to extract relevant data such as age, gender, phenotypic features that were observed and excluded in the patient, and onset information. This information is then used together with a programmatic template to generate a clinical narrative suitable for LLMs. The template first specifies the sex of the individual, the age of onset, and the age at last examination, and then lists the HPO terms that represent observed or excluded clinical features. If available, separate lists are included for different ages of examination (Figure 1). The software is implemented as a Java package. *phenopacket2prompt* is freely available on GitHub under an open source MIT license at https://github.com/monarch-initiative/phenopacket2prompt.

### Computational analysis of GPT-4 responses

The prompts generated as described above were then sent to GPT-4 using the OpenAI API to generate differential diagnoses. We constructed software, MALCO, to evaluate the performance of GPT-4 for the cases described above. For each case, the prompt was sent to GPT-4 (version gpt-4-turbo-2024–04-09) via the OpenAI API and the differential diagnosis as a ranked list in plain text was saved. Each item in the ranked list was converted to Mondo Disease Ontology terms using the Ontology Access Kit (OAK) and OntoGPT.^17^ For each item, the equivalent OMIM disease terms for the Mondo term were determined using mappings present in the Mondo ontology, and the item was marked as correct if Mondo term mapped to the correct OMIM identifier from the original phenopacket. For each case, the rank of the correct diagnosis, if present, was recorded. MALCO is freely available on GitHub under an open-source BSD 3-Clause License at https://github.com/monarch-initiative/malco. OAK is freely available on GitHub under an open-source Apache 2 license at https://github.com/INCATools/ontology-access-kit.

### Reporting

Reporting in this study followed Consolidated Reporting Guidelines for Prognostic and Diagnostic Machine Learning Modeling Studies.^18^

## Results

**Figure 2.**
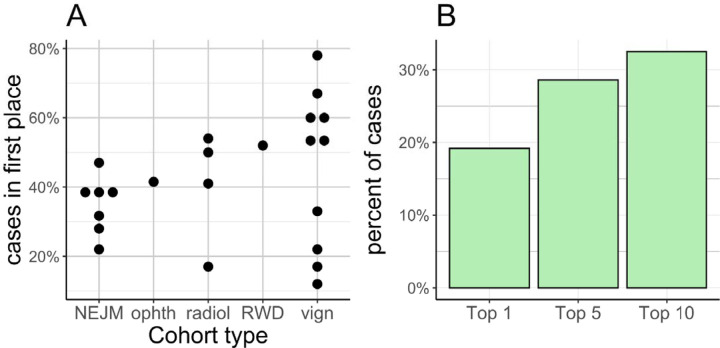
Accuracy of GPT-4 in differential diagnostic challenges. (A) Summary of performance in 23 previously published studies that reported the percentage of cases in which the correct diagnosis was placed at rank 1 by the LLM. Cohorts were derived from multiple sources including published clinical vignettes (vign), New England Journal of Medicine case reports or quizzes (NEJM), JAMA Ophthalmology Clinical Challenges (ophth), and case reports including clinical data and radiology reports in text form (radiol), and one cohort of real-world data (RWD; 6 patients). Details are available in Supplemental Table 1. (B) The percentage of cases of the current cohort of 5267 cases in which GPT-4 returned the correct diagnosis at rank 1 (Top 1), within the top 3 ranks (Top 3), or within the top 10 ranks (Top 10).

We identified 23 previous publications that evaluated the performance of LLMs on differential diagnostic challenges using text prompts (Supplemental Table 1). The reported performance varied widely, even for studies using the same input data such as the NEJM Case Studies (Figure 2A). We reasoned that the variability could be partially due to subjective decisions made as to whether an LLM response exactly matched the correct diagnosis. To mitigate this potential source of bias, we developed an approach to programmatically map responses of GPT-4 to terms from the Mondo ontology, which provides a comprehensive and standardized framework used for the classification of human diseases that integrates various disease classification systems, and thereby provides a unified approach to disease nomenclature.^19^ In particular, we used Mondo to merge groups of diseases for the purposes of evaluation (e.g., MONDO:0015229 Bardet-Biedl syndrome represents subtypes 1 through 22 of Bardet-Biedl syndrome).

We leveraged a collection of GA4GH phenopackets with data from 5267 individuals with 336 Mendelian or chromosomal diseases, which arrange data using ontology terms and structured fields. We programmatically generated prompts from the phenopackets using a standard template (all phenopackets and prompts are available as Supplemental File 1).

We presented GPT-4 with these prompts generated from GA4GH phenopackets. We asked GPT-4 to return a differential diagnosis as a list of disease names and recorded the rank of the correct diagnosis in these lists, if present. We then investigated whether GPT-4 returned the correct clinical diagnosis (e.g., Bardet-Biedl syndrome) rather than the original precise genetic diagnosis (e.g., BBS type 13) because no genetic information was used for this experiment. The mean reciprocal rank of the correct diagnosis was 0.24 (with the reciprocal of the MRR corresponding to a rank of 4.2), and the correct diagnosis was placed in rank 1 in 19.2% (1009/5264) of the cases, in the first 3 ranks in 28.6% (1503/5264), and in the first 10 ranks in 32.5% (1709/5264) (Figure 2). The API call to GPT-4 failed in three of the 5267 cases. These cases were omitted from analysis.

## Discussion

LLMs have demonstrated impressive performance on several medical tasks including knowledge retrieval, addressing patient questions, and summarizing key findings.^1^ Clinical decision support is an area of great potential promise, especially for rare disease medicine in which diagnostics tends to be challenging and many patients experience a diagnostic odyssey lasting several years before receiving a precise diagnosis. Previous studies that evaluate the performance of LLMs in this area have had relatively small sample sizes and have employed manual and subjective evaluation as to whether LLM responses exactly match the correct diagnosis. No published study has focused specifically on rare disease.

We have analyzed a dataset of over 5000 structured representations of clinical cases that were transformed into phenopackets using the same programmatic template. The analysis was conducted using GPT-4 and results were evaluated programmatically by matching GPT responses to standard ontology codes for diseases. Our analysis thereby minimizes subjective choices and provides a realistic estimate of the expected performance of GPT over a broad range of rare diseases. We cannot directly compare our results to those of previous studies, which did not analyze cohorts of individuals with rare disease. Our result is similar to the lower range of previously reported studies, which may be related to the difficulties in diagnosing rare disease.^20^

## Limitations

Limitations of our study include the fact that the representation of the clinical phenotypes with HPO terms in the phenopackets may have been incomplete or inaccurate. Additionally, the description of the clinical features in the publications from which the phenopackets were derived may have been incomplete. We did not undertake fine-tuning or prompt-tuning in this analysis; these procedures may increase performance on specific clinical decision-making tasks.^21^ Therefore, it may be possible to increase overall performance, and it is possible that performance may improve with future versions of GPT or with specialized LLMs. However, the approach we present here is similar to that of the 23 previous studies summarized in Figure 2A and supplemental Table 1.

## Conclusions

We have presented the largest reported study on the differential diagnostic capabilities of GPT-4, the LLM that is the current best in class for a variety of medical applications. Our analysis approach was designed to minimize variability and subjective choices in evaluation, and thereby provides a realistic estimate of the performance of GPT in rare-disease differential diagnostics.

## Supplementary Material

Supplement 1

## References

[1] Singhal K, Azizi S, Tu T, Large language models encode clinical knowledge. Nature. 2023;620(7972):172–180. 37438534 10.1038/s41586-023-06291-2 PMC10396962

[2] Moor M, Banerjee O, Abad ZSH, Foundation models for generalist medical artificial intelligence. Nature. 2023;616(7956):259–265. 37045921 10.1038/s41586-023-05881-4

[3] Kanjee Z, Crowe B, Rodman A. Accuracy of a Generative Artificial Intelligence Model in a Complex Diagnostic Challenge. JAMA. Published online June 15, 2023. doi:10.1001/jama.2023.8288 PMC10273128 37318797

[4] Abdullahi T, Singh R, Eickhoff C. Learning to Make Rare and Complex Diagnoses With Generative AI Assistance: Qualitative Study of Popular Large Language Models. JMIR Med Educ. 2024;10:e51391. 38349725 10.2196/51391 PMC10900078

[5] Ríos-Hoyo A, Shan NL, Li A, Pearson AT, Pusztai L, Howard FM. Evaluation of large language models as a diagnostic aid for complex medical cases. Front Med. 2024;11:1380148. 10.3389/fmed.2024.1380148 PMC11222590 38966538

[6] Chiu WHK, Ko WSK, Cho WCS, Hui SYJ, Chan WCL, Kuo MD. Evaluating the Diagnostic Performance of Large Language Models on Complex Multimodal Medical Cases. J Med Internet Res. 2024;26:e53724. 38739441 10.2196/53724 PMC11130768

[7] Scott NS, Thomas SS, DeFaria Yeh D, Fox AS, Smith RN. Case 2–2021: A 26-Year-Old Pregnant Woman with Ventricular Tachycardia and Shock. N Engl J Med. 2021;384(3):272–282. 33471980 10.1056/NEJMcpc2027086

[8] Reese JT, Danis D, Caufield JH, On the limitations of large language models in clinical diagnosis. medRxiv. Published online February 26, 2024. doi:10.1101/2023.07.13.23292613

[9] Dua A, Sutphin PD, Siedner MJ, Moran J. Case 16–2021: A 37-Year-Old Woman with Abdominal Pain and Aortic Dilatation. N Engl J Med. 2021;384(21):2054–2063. 34042393 10.1056/NEJMcpc2100278

[10] Ueda D, Mitsuyama Y, Takita H, ChatGPT’s Diagnostic Performance from Patient History and Imaging Findings on the Diagnosis Please Quizzes. Radiology. 2023;308(1):e231040. 37462501 10.1148/radiol.231040

[11] Milad D, Antaki F, Milad J, Assessing the medical reasoning skills of GPT-4 in complex ophthalmology cases. Br J Ophthalmol. Published online February 16, 2024. doi:10.1136/bjo-2023-325053 38365427

[12] Vandeborne L, van Overbeeke E, Dooms M, De Beleyr B, Huys I. Information needs of physicians regarding the diagnosis of rare diseases: a questionnaire-based study in Belgium. Orphanet J Rare Dis. 2019;14(1):99. 31054581 10.1186/s13023-019-1075-8 PMC6500578

[13] Haendel M, Vasilevsky N, Unni D, How many rare diseases are there? Nat Rev Drug Discov. 2020;19(2):77–78. 32020066 10.1038/d41573-019-00180-y PMC7771654

[14] Jacobsen JOB, Baudis M, Baynam GS, The GA4GH Phenopacket schema defines a computable representation of clinical data. Nat Biotechnol. 2022;40(6):817–820. 35705716 10.1038/s41587-022-01357-4 PMC9363006

[15] Danis D, Bamshad MJ, Bridges Y, A corpus of GA4GH Phenopackets: case-level phenotyping for genomic diagnostics and discovery. bioRxiv. Published online May 29, 2024. doi:10.1101/2024.05.29.24308104 PMC11564936 39394689

[16] Harada Y, Sakamoto T, Sugimoto S, Shimizu T. Longitudinal Changes in Diagnostic Accuracy of a Differential Diagnosis List Developed by an AI-Based Symptom Checker: Retrospective Observational Study. JMIR Form Res. 2024;8:e53985. 38758588 10.2196/53985 PMC11143391

[17] Harry Caufield J, Hegde H, Emonet V, Structured prompt interrogation and recursive extraction of semantics (SPIRES): A method for populating knowledge bases using zero-shot learning. arXiv [csAI]. Published online April 5, 2023. http://arxiv.org/abs/2304.02711 10.1093/bioinformatics/btae104 PMC10924283 38383067

[18] Klement W, El Emam K. Consolidated Reporting Guidelines for Prognostic and Diagnostic Machine Learning Modeling Studies: Development and Validation. J Med Internet Res. 2023;25:e48763. 37651179 10.2196/48763 PMC10502599

[19] Shefchek KA, Harris NL, Gargano M, The Monarch Initiative in 2019: an integrative data and analytic platform connecting phenotypes to genotypes across species. Nucleic Acids Res. 2020;48(D1):D704–D715. 31701156 10.1093/nar/gkz997 PMC7056945

[20] Faye F, Crocione C, Anido de Peña R, Time to diagnosis and determinants of diagnostic delays of people living with a rare disease: results of a Rare Barometer retrospective patient survey. Eur J Hum Genet. Published online May 16, 2024. doi:10.1038/s41431-024-01604-z PMC11369105 38755315

[21] Hager P, Jungmann F, Holland R, Evaluation and mitigation of the limitations of large language models in clinical decision-making. Nat Med. Published online July 4, 2024. doi:10.1038/s41591-024-03097-1 PMC11405275 38965432

