# Systematic benchmarking demonstrates large language models have not reached the diagnostic accuracy of traditional rare-disease decision support tools

**DOI:** 10.1101/2024.07.22.24310816

**Published:** 2024-11-07

**Authors:** Justin T Reese, Leonardo Chimirri, Yasemin Bridges, Daniel Danis, J Harry Caufield, Kyran Wissink, Julie A McMurry, Adam SL Graefe, Elena Casiraghi, Giorgio Valentini, Julius OB Jacobsen, Melissa Haendel, Damian Smedley, Christopher J Mungall, Peter N Robinson

**Affiliations:** 1) Division of Environmental Genomics and Systems Biology, Lawrence Berkeley National Laboratory, Berkeley, CA, USA,; 2) Monarch Initiative; 3) Berlin Institute of Health at Charite Universitaetsmedizin Berlin, Berlin, Germany; 4) William Harvey Research Institute, Barts and The London School of Medicine and Dentistry, Queen Mary University of London, London, UK; 5) University of North Carolina at Chapel Hill, Chapel Hill, NC, USA; 6) Division of Environmental Genomics and Systems Biology, Lawrence Berkeley National Laboratory, Berkeley, CA, USA; 7) AnacletoLab, Dipartimento di Informatica, Università degli Studi di Milano, Milano, Italy; 8) ELLIS-European Laboratory for Learning and Intelligent Systems; 9) The Jackson Institute for Genomic Medicine, 10 Discovery Drive, Farmington CT 06032, USA

## Abstract

Large language models (LLMs) show promise in supporting differential diagnosis, but their performance is challenging to evaluate due to the unstructured nature of their responses. To assess the current capabilities of LLMs to diagnose genetic diseases, we benchmarked these models on 5,213 case reports using the Phenopacket Schema, the Human Phenotype Ontology and Mondo disease ontology. Prompts generated from each phenopacket were sent to three generative pretrained transformer (GPT) models. The same phenopackets were used as input to a widely used diagnostic tool, Exomiser, in phenotype-only mode. The best LLM ranked the correct diagnosis first in 23.6% of cases, whereas Exomiser did so in 35.5% of cases. While the performance of LLMs for supporting differential diagnosis has been improving, it has not reached the level of commonly used traditional bioinformatics tools. Future research is needed to determine the best approach to incorporate LLMs into diagnostic pipelines.

LLMs are general-purpose artificial intelligence models that can be applied to numerous tasks across diverse domains. It has been proposed that LLMs may be useful for many clinical tasks, such as charting, medication review, or clinical trial matching.^1,2^ For differential diagnostic support, LLMs can be prompted to return a ranked list of potential diagnoses based on narrative text describing a patient’s features.^3^

We identified 36 previous publications that evaluated the performance of LLMs on differential diagnostic challenges using text prompts (Supplemental Table 1). Thirty-four of 36 publications we identified involved human curation, usually by physicians, to compare the response of LLMs (most frequently, OpenAI’s GPT models) to the correct diagnosis recorded in the original source (Details and citations in Supplemental Figure S1 and Supplemental Table S1). These studies analyzed cohorts of between 6 and 9681 cases (median 78), often from published vignettes intended for medical education, such as the *Case Studies* of New England Journal of Medicine,^3–6^ the *Diagnosis Please* quizzes from the journal Radiology,^7^ and JAMA Ophthalmology *Clinical Challenges*.^8^ The reported performance varied widely, even for studies using the same input data such as the NEJM Case Studies (Supplemental Figure 1). We reasoned that the variability could be partially due to subjective judgements as to whether an LLM response exactly matched the correct diagnosis.

Although conceptually simple, this step requires that the curator have specialized medical knowledge of the disease in question. For instance, in case Case 2–2021 of the New England Journal of Medicine Case Record series that has been used by multiple groups to assess LLM performance,^3^ the final diagnosis was given as “pregnancy-associated myocardial infarction, probably due to spontaneous coronary-artery dissection”.^9^ In our analysis of this case,^10^ GPT-4 returned answers including “peripartum cardiomyopathy” and “heart failure secondary to severe pre-eclampsia,” both of which are cardiovascular complications following delivery, but neither of which is correct. As a second example, in case 16–2021, the final diagnosis was “*Staphylococcus aureus* bacteremia and infection of a vascular graft”.^11^ In our analysis of this case, the diagnosis at rank 4, “infective endocarditis affecting the aortic valve and causing referred abdominal pain,” was similar to the correct diagnosis. In a study on the NEJM cases, two scorers agreed on only 66% of scores in 80 NEJM cases.^3^ Therefore, the practical utility of LLM analysis may be limited by the varying ability of human users to interpret the responses, and manual curation that may influence the measured performance of LLMs. For this reason, we concluded that a computational approach to evaluate the responses of GPT-4 by assigning them to specific disease entities (Mondo ontology terms) would provide a more realistic assessment of the performance of GPT-4 for diagnostic use.

Two recent LLM studies^12,13^ focus on diagnosing rare genetic disease (RD), an area of great need. Rare diseases, collectively, are not rare: over 10,000 rare diseases have been identified to date, together affecting between 3.5% and 8% of the population. Furthermore, affected individuals often experience a diagnostic odyssey lasting 5–7 years.^14,15^ One of the studies of LLMs for RD diagnostics requested the LLMs to return gene symbols rather than disease names or codes,^12^ which obviates the need to manually check for equivalence of potentially synonymous disease names; however, LLMs are known to frequently return erroneous gene and ontology identifiers (a form of hallucination).^16^ This study of the performance of several LLMs on 276 published case reports showed that GPT-4 had the best performance, placing the correct diagnosis of 13.9% of cases within the top ten ranked diagnoses. However, there are several potential shortcomings with this approach. Predicting disease genes is arguably more challenging than predicting diseases, because one gene may be associated with many diseases, and the same (clinically defined) disease may be associated with many genes. For example, the authors included genes like *LMNA*, which is linked to 11 distinct diseases in Online Mendelian Inheritance in Man (OMIM), and *IFT172*, which is associated with three diseases, of which one (Bardet-Biedl syndrome) is a genetically complex condition that can result from pathogenic variants in any of at least 22 different genes. In this study, the correct gene was placed in the top ten ranked candidates in 11.7% of cases; the rank-1 performance was not reported.

Another recent study analyzed prompts for 63 genetic conditions and compared the performance using medical and lay language.^13^ The 63 cases were created with 2–5 characteristic phenotype terms each and were described by the authors as “textbook-like descriptions.” This work involved a similar manual assessment step to determine if the response of the LLM was correct; for instance, in one case, the authors assigned “Riley-Day syndrome” to its synonym “hereditary sensory and autonomic neuropathy, type III”, and reported that some LLM responses required additional discussion among the graders. Among the LLMs to date, GPT models have been shown to have the best general performance in differential diagnosis (see references in Supplemental Table 1).

To provide an estimate of the performance of LLMs, we assembled data from 5213 individuals with a previously solved diagnosis; each case was structured as a phenopacket^17^, a Global Alliance for Genomics and Health (GA4GH) and ISO standard. Each phenopacket provides a structured representation of Human Phenotype Ontology (HPO) terms representing the signs, symptoms, abnormal imaging findings and laboratory test results that were observed or excluded in a single patient. Collectively, the diagnoses spanned 378 unique Mendelian or chromosomal diseases; the phenopacket encodes these as (OMIM)^18^ identifiers. The identified causal variants, while part of the phenopacket, were not used in the current analysis. We programmatically generated prompts from the phenopackets using a standard template (all phenopackets and prompts are available via Zenodo - see data availability section).

We analyzed the same 5213 cases to compare performance between GPT models and a traditional bioinformatics tool, Exomiser, which was developed by the Monarch Initiative. Exomiser was shown to be the best performing diagnostic tool on 100,000 Genomes Project data^19^ and is widely used in diagnostic labs, large-scale disease sequencing projects and national healthcare services such as the UK’s Genomic Medicine Service. For the comparison herein, we used Exomiser in ‘phenotype only’ mode. To mitigate the potential of bias implied by manual comparison of LLM responses to the expected correct result, we developed an approach to programmatically map (i.e. ‘ground’) responses of the LLM to terms from the Monarch Initiative’s Mondo disease ontology (https://github.com/monarch-initiative/mondo), which provides a comprehensive and standardized framework used for the classification of human diseases that integrates various disease classification systems, and thereby provides a unified approach to disease nomenclature.^20^ For genetically heterogeneous diseases such as geleophysic dysplasia, we used Mondo to “roll up” groups of diseases (e.g., MONDO:0000127 geleophysic dysplasia subtypes 1 through 3 of geleophysic dysplasia).

We presented the LLM with these prompts generated from phenopackets (Figure 1; Supplemental Tables S2–S6). We prompted the model to return a differential diagnosis as a list of disease names and determined the rank of the correct diagnosis in these lists, if present. Our strategy employs the Mondo ontology to count clinical diagnoses (e.g., geleophysic dysplasia) as correct, in addition to the the original precise genetic diagnosis (e.g., geleophysic dysplasia 2) since no genetic information was provided to any diagnostic tool in this experiment. We used the PhEval evaluation framework^21^ to rigorously benchmark results.

The diagnostic accuracy of Exomiser was greater than any of the LLMs tested. Exomiser placed the correct diagnosis in the first rank in 35.5% of cases, compared with 23.6% for the best performing LLM (o1 preview) (Figure 2). Similarly, Exomiser placed the correct diagnosis in the top 3 ranks of the differential diagnosis in 46.3% of cases and in the top 10 ranks in 58.5% of cases, compared with 31.2% in the top 3 ranks and 36.8% in the top 10 ranks for the best performing LLM (o1 preview).

Previous studies that evaluated the performance of LLMs in the area of decision support for differential diagnosis have had relatively small sample sizes and have employed manual and subjective evaluation of whether LLM responses match the correct diagnosis. Our analysis uses an ontology-based strategy that eliminates subjective choices as to whether an unstructured response returned by an LLM contains the correct diagnosis and provides a realistic estimate of the expected performance of LLMs over a broad range of rare diseases. In our analysis, the best performing LLM (o1 preview) was able to place the correct diagnosis in the first ten ranks in 36.8% of the 5213 cases, much higher than in the study using gene symbols (12.11%); the performance was worse than for the 63 textbook-like disease descriptions (81%–89% top-10 accuracy). In our study, the performance of Exomiser was substantially better than that of the best LLM using only phenotype data. Additionally, Exomiser is designed to work with both phenotypic and whole-exome or whole-genome data; on 4877 molecularly diagnosed cases from the 100,000 Genome Project, Exomiser prioritized the correct gene in the top, top 3, and top 10 ranked candidates 82.6%, 91.3%, and 93.6% of the time.^22^ We did not test the utility of providing the LLM with genomic information in the current analysis; including genetic information would require additional measures to ensure privacy if used for actual patients.

Limitations of our study include the fact that the representation of the clinical phenotypes with HPO terms in the phenopackets may have been incomplete or inaccurate. Additionally, the description of the clinical features in the publications from which the phenopackets were derived may have been incomplete. We did not undertake fine-tuning or prompt-tuning in this analysis; these procedures may increase performance on specific clinical decision-making tasks.^23^ A recent study that employs a likelihood ratio method combined with retrieval augmented prompt generation, followed by querying of GPT-3.5-turbo, showed a top-ten performance of 69.33% (165 out of 238 cases) for the true diagnoses in the top 10; however, the same cases were used to train the model, so that the expected performance on new cases and diseases remains to be characterized.^16^

In conclusion, we present the largest reported study on the differential diagnostic capabilities of the GPT family of LLMs, the LLM that is the current best in class for a variety of medical applications. Our analysis approach was designed to minimize variability and subjective choices in evaluation, and thereby provides a realistic estimate of the performance of GPT in rare-disease differential diagnostics, and shows that the performance of GPT for RD differential diagnostic support is currently clearly inferior to that of a commonly used traditional bioinformatics tool, Exomiser. Future work will be required to determine how to design and integrate LLMs into diagnostic pipelines for RD genomic diagnostics.

## Online Methods

### Study design and data

We evaluated the performance of LLMs in differential diagnosis using 5213 computational case reports formatted as GA4GH phenopackets taken from the phenopacket-store repository (version 0.14).^24^ The case reports describe 378 Mendelian and chromosomal diseases associated with 336 genes. Each phenopacket contains information derived from published case or cohort reports from a total of 726 different publications. The diagnosis indicated in the original publication was recorded but not used in generating the prompt. A total of 2975 distinct HPO terms were used, with an average of 16 HPO terms per case.

This study was deemed exempt from local IRB approval as it did not meet the criteria for human subjects research, because the cases were curated from publicly available articles.

### Retrieval of relevant literature

A search was conducted in PubMed to retrieve articles that describe the use of LLMs for differential diagnostics. The search string was:

(“GPT”[Title/Abstract] OR “LLM”[Title/Abstract] OR “Large language model”[Title/Abstract])
AND
(“differential diagnostics”[Title/Abstract] OR “differential diagnosis”[Title/Abstract])
AND
(“2023”[Date - Create] : “3000”[Date - Create])

This search was performed on Oct 18, 2024, and returned 63 articles.

This list was further refined by selecting only articles that described the application of one or more LLMs to perform differential diagnostic analysis on a cohort of clinical cases. Further, for better comparability, we only retained publications in which the rate of placing the correct diagnosis in rank 1 was reported. For instance, we omitted one publication because only the rate of the correct diagnosis in the top three candidates was reported.^25^ The reference lists of chosen publications were scanned to identify additional articles. A full list of included articles is provided in Supplemental Table 1.

### Computational generation of prompts for LLMs

We constructed software, *phenopacket2prompt*, to convert case data in GA4GH phenopacket format to prompts suitable for use with LLMs to generate differential diagnoses. By parsing phenopackets, the software extracts relevant data such as age, gender, phenotypic features that were observed and excluded in the patient, and onset information. This information is then used together with a programmatic template to generate a clinical narrative suitable for LLMs. The template first specifies the sex of the individual, the age of onset, and the age at last examination, and then lists the HPO terms that represent observed or excluded clinical features. If available, separate lists are included for different ages of examination (Supplemental Figure S7). The software is implemented as a command-line Java application and is freely available on GitHub under an open source MIT license at https://github.com/monarch-initiative/phenopacket2prompt.

### Generating differential diagnoses

#### Exomiser

Exomiser was used to generate differential diagnoses as follows. Exomiser version 14.0.1 was downloaded from https://github.com/exomiser/Exomiser/releases/download/14.0.1/exomiser-cli-14.0.1-distribution.zip, and the 2406 version of the Exomiser data release was downloaded from: https://data.monarchinitiative.org/exomiser/data/2406_phenotype.zip https://data.monarchinitiative.org/exomiser/data/2406_hg19.zip Exomiser was applied in phenotype only mode to generate a differential diagnosis (comprising ranked lists of OMIM or Orphanet IDs) for each phenopacket.

#### LLMs (o1 mini, o1 preview, and GPT-4o)

The prompts generated as described above were provided to o1-preview (version o1-preview-2024–09-12), o1 mini (version o1-mini-2024–09-12), and GPT-4o (version gpt-4o-2024–08-06) to generate differential diagnoses. For each case, each item in the differential diagnosis generated by the LLM was converted to Mondo Disease Ontology terms as follows: first, items that matched exactly to a Mondo disease label or synonym were assigned Mondo terms using the Ontology Access Kit (OAK), and the remaining items were assigned the best matching Mondo term above an empirically determined threshold using curategpt (https://github.com/monarch-initiative/curategpt). 94.0% and 95.6% of items in the differential diagnoses were assigned Mondo terms (see supplemental data).

### Scoring differential diagnoses

**Figure 1.**
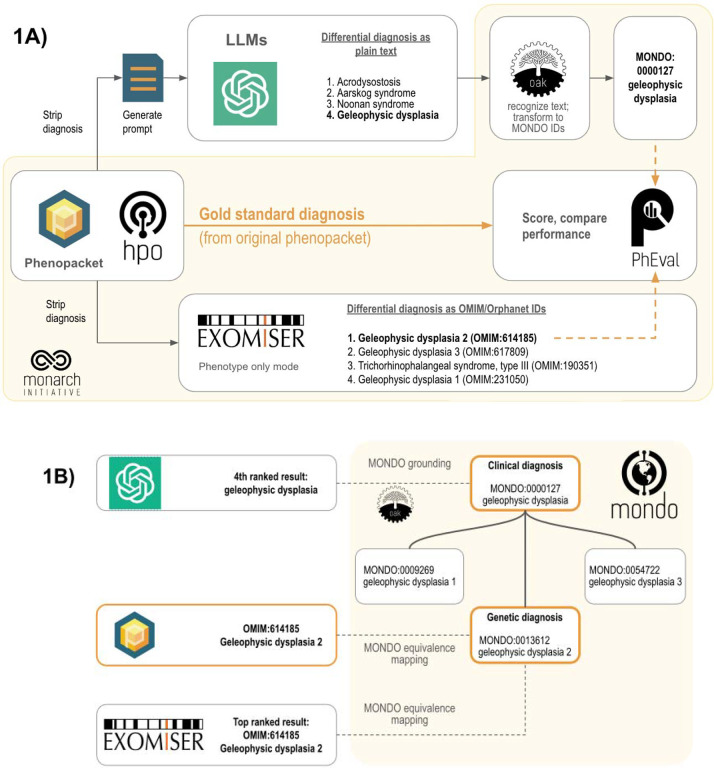
Workflow for comparing the accuracy of differential diagnoses from Exomiser and LLMs. Each phenopacket in the cohort of 5213 cases is used to generate a differential diagnosis using LLMs or Exomiser. **(1A)** The phenopacket is used to generate a prompt containing case data (age, gender, observed and excluded phenotypic features, and the onset of phenotypic features, if present), and each item in response from the LLMs is converted to Mondo disease identifiers using concept recognition software (OAK). Exomiser uses the same phenopacket to generate a differential diagnosis comprising OMIM or Orphanet disease identifiers from the Exomiser dataset. The rank of the correct diagnosis, if present, is determined by comparing them to the gold standard diagnosis from the phenopacket using an ontology-based strategy. **(1B)** Ontology-based strategy for identifying correct diagnoses (orange) using Mondo. Items in the differential diagnoses from LLM (typically clinical diagnoses, e.g. geleophysic dysplasia) are grounded to Mondo, and items from Exomiser (typically genetic diagnoses, e.g. geleophysic dysplasia 2) and the correct diagnosis from the phenopacket are aligned to Mondo using equivalence mappings in Mondo. An item in a differential diagnosis is considered correct if it matches the diagnosis from the phenopacket (OMIM:614185 geleophysic dysplasia 2), matches an equivalent Mondo disease (MONDO:0013612 geleophysic dysplasia 2), or matches a more general Mondo disease with a descendant that is a correct diagnosis (MONDO:0000127 geleophysic dysplasia).

For each case, each item in the differential diagnosis was scored as correct or incorrect using the Ontology Access Kit (OAK, https://github.com/INCATools/ontology-access-kit) as follows. An item from the differential diagnosis was considered correct if the disease identifier for the diagnosis matched the identifier for gold standard diagnosis from the case report exactly, or was mapped as equivalent to the identifier of the gold standard diagnosis in Mondo, or if the gold standard diagnosis was a close descendant of the LLM’s diagnosis identifier in Mondo (Figure 1). Thus, more general items such as *geleophysic dysplasia 2* (MONDO:0013612) are considered as correct diagnoses for more specific items that are subsumed by the general one, such as *geleophysic dysplasia* (MONDO:0000127). For each case, the rank of the correct diagnosis, if present, was recorded. The grounding and scoring software is freely available on GitHub under an open-source BSD 3-Clause License at https://github.com/monarch-initiative/pheval.llm. OAK is freely available on GitHub under an open-source Apache 2 license.

### Reporting

Reporting in this study followed Consolidated Reporting Guidelines for Prognostic and Diagnostic Machine Learning Modeling Studies.^26^

## Supplementary Material

Supplement 1

## Figures and Tables

**Figure 2.**
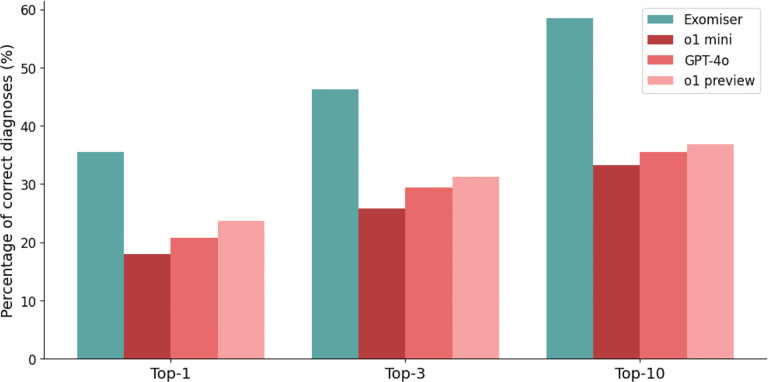
Accuracy of Exomiser, o1 mini, GPT-4o, and o1 preview in differential diagnostic challenges. The bar chart shows the percentage of cases of the current cohort of 5213 cases in which Exomiser (teal), o1 mini (red), GPT-4o (coral), and o1 preview (pink) returned a correct diagnosis (see Methods) at rank 1 (Top 1), within the top 3 (Top 3), or within the top 10 ranks (Top 10).

## Data Availability

Computational representations of published individual-level clinical data in the form of GA4GH phenopackets and prompts intended for use with LLMs. The dataset, prompts, and differential diagnoses generated by LLMs and Exomiser are available on Zenodo: https://zenodo.org/records/14008477. All data collected for the study is available to all under an open source CC-BY License.

## References

[1] Singhal K. Large language models encode clinical knowledge. Nature 620, 172–180 (2023). 37438534 10.1038/s41586-023-06291-2 PMC10396962

[2] Moor M. Foundation models for generalist medical artificial intelligence. Nature 616, 259–265 (2023). 37045921 10.1038/s41586-023-05881-4

[3] Kanjee Z., Crowe B. & Rodman A. Accuracy of a Generative Artificial Intelligence Model in a Complex Diagnostic Challenge. JAMA (2023) doi:10.1001/jama.2023.8288. PMC10273128 37318797

[4] Abdullahi T., Singh R. & Eickhoff C. Learning to Make Rare and Complex Diagnoses With Generative AI Assistance: Qualitative Study of Popular Large Language Models. JMIR Med Educ 10, e51391 (2024). 38349725 10.2196/51391 PMC10900078

[5] Ríos-Hoyo A. Evaluation of large language models as a diagnostic aid for complex medical cases. Front. Med. 11, 1380148 (2024). 10.3389/fmed.2024.1380148 PMC11222590 38966538

[6] Chiu W. H. K. Evaluating the Diagnostic Performance of Large Language Models on Complex Multimodal Medical Cases. J. Med. Internet Res. 26, e53724 (2024). 38739441 10.2196/53724 PMC11130768

[7] Ueda D. ChatGPT’s Diagnostic Performance from Patient History and Imaging Findings on the Diagnosis Please Quizzes. Radiology 308, e231040 (2023). 37462501 10.1148/radiol.231040

[8] Milad D. Assessing the medical reasoning skills of GPT-4 in complex ophthalmology cases. Br. J. Ophthalmol. (2024) doi:10.1136/bjo-2023-325053. 38365427

[9] Scott N. S., Thomas S. S., DeFaria Yeh D., Fox A. S. & Smith R. N. Case 2–2021: A 26-Year-Old Pregnant Woman with Ventricular Tachycardia and Shock. N. Engl. J. Med. 384, 272–282 (2021). 33471980 10.1056/NEJMcpc2027086

[10] Reese J. T. On the limitations of large language models in clinical diagnosis. medRxiv (2024) doi:10.1101/2023.07.13.23292613.

[11] Dua A., Sutphin P. D., Siedner M. J. & Moran J. Case 16–2021: A 37-Year-Old Woman with Abdominal Pain and Aortic Dilatation. N. Engl. J. Med. 384, 2054–2063 (2021). 34042393 10.1056/NEJMcpc2100278

[12] Kim J., Wang K., Weng C. & Liu C. Assessing the utility of large language models for phenotype-driven gene prioritization in the diagnosis of rare genetic disease. Am. J. Hum. Genet. 111, 2190–2202 (2024). 39255797 10.1016/j.ajhg.2024.08.010 PMC11480789

[13] Flaharty K. A. Evaluating large language models on medical, lay-language, and self-reported descriptions of genetic conditions. Am. J. Hum. Genet. 111, 1819–1833 (2024). 39146935 10.1016/j.ajhg.2024.07.011 PMC11393706

[14] Vandeborne L., van Overbeeke E., Dooms M., De Beleyr B. & Huys I. Information needs of physicians regarding the diagnosis of rare diseases: a questionnaire-based study in Belgium. Orphanet J. Rare Dis. 14, 99 (2019). 31054581 10.1186/s13023-019-1075-8 PMC6500578

[15] Haendel M. How many rare diseases are there? Nat. Rev. Drug Discov. 19, 77–78 (2020). 32020066 10.1038/d41573-019-00180-y PMC7771654

[16] Yang J., Shu L., Duan H. & Li H. RDguru: A conversational intelligent agent for rare diseases. IEEE J. Biomed. Health Inform. PP, (2024). 10.1109/JBHI.2024.3464555 39298307

[17] Jacobsen J. O. B. The GA4GH Phenopacket schema defines a computable representation of clinical data. Nat. Biotechnol. 40, 817–820 (2022). 35705716 10.1038/s41587-022-01357-4 PMC9363006

[18] Amberger J. S., Bocchini C. A., Schiettecatte F., Scott A. F. & Hamosh A. OMIM.org: Online Mendelian Inheritance in Man (OMIM^®^), an online catalog of human genes and genetic disorders. Nucleic Acids Res. 43, D789–98 (2015). 25428349 10.1093/nar/gku1205 PMC4383985

[19] 100,000 Genomes Project Pilot Investigators 100,000 Genomes Pilot on Rare-Disease Diagnosis in Health Care - Preliminary Report. N. Engl. J. Med. 385, 1868–1880 (2021). 34758253 10.1056/NEJMoa2035790 PMC7613219

[20] Shefchek K. A. The Monarch Initiative in 2019: an integrative data and analytic platform connecting phenotypes to genotypes across species. Nucleic Acids Res. 48, D704–D715 (2020). 31701156 10.1093/nar/gkz997 PMC7056945

[21] BridgesY. S. Towards a standard benchmark for variant and gene prioritisation algorithms: PhEval - Phenotypic inference Evaluation framework. bioRxiv 2024.06.13.598672 (2024) doi:10.1101/2024.06.13.598672.40121479

[22] Jacobsen J. O. B. Phenotype-driven approaches to enhance variant prioritization and diagnosis of rare disease. Hum. Mutat. 43, 1071–1081 (2022). 35391505 10.1002/humu.24380 PMC9288531

[23] Hager P. Evaluation and mitigation of the limitations of large language models in clinical decision-making. Nat. Med. (2024) doi:10.1038/s41591-024-03097-1. PMC11405275 38965432

[24] Danis D. A corpus of GA4GH Phenopackets: case-level phenotyping for genomic diagnostics and discovery. bioRxiv (2024) doi:10.1101/2024.05.29.24308104. PMC11564936 39394689

[25] Harada Y., Sakamoto T., Sugimoto S. & Shimizu T. Longitudinal Changes in Diagnostic Accuracy of a Differential Diagnosis List Developed by an AI-Based Symptom Checker: Retrospective Observational Study. JMIR Form Res 8, e53985 (2024). 38758588 10.2196/53985 PMC11143391

[26] Klement W. & El Emam K. Consolidated Reporting Guidelines for Prognostic and Diagnostic Machine Learning Modeling Studies: Development and Validation. J. Med. Internet Res. 25, e48763 (2023). 37651179 10.2196/48763 PMC10502599

