# Supplementary material for: Evaluation of the Diagnostic Accuracy of GPT-4 in Five Thousand Rare Disease Cases
Source: medRxiv. 2024 Jul 22:2024.07.22.24310816. Preprint. [Version 1] doi: 10.1101/2024.07.22.24310816 (PMC11302616; doi:10.1101/2024.07.22.24310816)
Supplement: Supplementary file 1 [file media-1.pdf]

author	size	rank 1	category	LLMs tested	evaluation	access
Hirosawa et al. (2023) [1]	30	53%	vignette	GPT-3.5	manual	Chatbox
Kanjee et al. (2023) [2]	70	39%	NEJM	GPT-4	manual	Chatbox
Hirosawa et al. (2023) [3]	52	60%	vignette	GPT-4	manual	Chatbox
Ueda et al. (2023) [4]	313	54%	radiology	GPT-4	manual	Chatbox
Rao et al. (2023)[5]	36	60%	vignette	GPT-3.5	manual	Chatbox
Shea et al. (2023) [6]	6	67%	vignette	GPT-4	manual	API
Shikino et al. (2024) [7]	25	12%	vignette	GPT-4	manual	Chatbox
Horiuchi et al. (2024) [8]	32	22%	vignette	GPT-4	manual	Chatbox
Horiuchi et al. (2024) [9]	100	50%	radiology	GPT-4	manual	Chatbox
Milad et al. (2024) [10]	422	42%	ophthalmology	GPT-4	manual	API
Abdullahi et al. (2024)[11]	45	47%	NEJM	Bard,GPT-3.5, GPT-4	manual	Chatbox
Kikuchi et al. (2024) [12]	115	41%	radiology	GPT-3.5,GPT-4	manual	Chatbox
Ríos-Hoyo et al. (2024)[13]	75	22%	NEJM	GPT-3.5,GPT-4	manual	Chatbox
Krusche et al. (2024) [14]	132	33%	vignette	GPT-4	manual	Chatbox
Rau et al. (2024)[15]	50	78%	vignette	GPT-4	manual	API
Chiu et al. (2024) [16]	104	32%	NEJM	Bard,Claude 2, GPT-4	manual	Chatbox
Barile et al. (2024) [17]	100	17%	vignette	GPT-3.5	manual	Chatbox
Li et al. (2024) [18]	287	17%	radiology	GPT-3.5, GPT-4	manual	Chatbox
Zandi et al. (2024) [19]	40	54%	vignette	GPT-4, Bard	manual	Chatbox
Luk et al. (2024) [20]	81	38%	NEJM	GPT-3.5, GPT-4	manual	Chatbox
Tenner et al. (2024) [21]	40	28%	NEJM	GPT-3.5	manual	Chatbox
Savage et al. (2024) [22]	310	38%	NEJM	GPT-3.5, GPT-4	manual	API
Koga et al. (2024) [23]	25	52%	RWD	GPT-3.5, GPT-4, Bard	manual	Chatbox

Table 1: **Summary of published evaluations of the performance of LLMs in differential diagnosis**. The meaning of the columns is as follows. **size**: The number of case reports (patients) evaluated. **rank 1**. The percentage of cases in which the correct diagnosis was placed at rank 1 by the LLM (for articles in which multiple LLMs were assessed, the best performance is indicated here). **category**: Vignette: A clinical vignette was derived from a published case report. NEJM: The prompt was derived from the New England Journal of Medicine case reports or quizes. opthalmology. The prompt was derived from JAMA Ophthalmology Clinical Challenges. radiology.The prompt was derived from sources such as the Diagnosis Please quizzes in Radiology, American Journal of Neuroradiology Case of the Week. **evaluation**: manual means that the authors evaluated the responses of the LLMs by hand. **Access**: Chatbox: The authors entered the prompts via a webinterface such as GhatGPT; API: The prompts were sent to the LLM programmatically.

References

[1] Takanobu Hirosawa, Yukinori Harada, Masashi Yokose, Tetsu Sakamoto, Ren Kawamura, and Taro Shimizu. Diagnostic accuracy of differential-diagnosis lists generated by generative pretrained transformer 3 chatbot for clinical vignettes with common chief complaints: A pilot study. *International journal of environmental research and public health*, 20, February 2023.

[2] Zahir Kanjee, Byron Crowe, and Adam Rodman. Accuracy of a generative artificial intelligence model in a complex diagnostic challenge. *JAMA*, 330:78–80, July 2023.

[3] Takanobu Hirosawa, Ren Kawamura, Yukinori Harada, Kazuya Mizuta, Kazuki Tokumasu, Yuki Kaji, Tomoharu Suzuki, and Taro Shimizu. Chatgpt-generated differential diagnosis lists for complex case-derived clinical vignettes: Diagnostic accuracy evaluation. *JMIR medical informatics*, 11:e48808, October 2023.

[4] Daiju Ueda, Yasuhito Mitsuyama, Hirotaka Takita, Daisuke Horiuchi, Shannon L. Walston, Hiroyuki Tatekawa, and Yukio Miki. Chatgpt's diagnostic performance from patient history and imaging findings on the diagnosis please quizzes. *Radiology*, 308:e231040, July 2023.

[5] Arya Rao, Michael Pang, John Kim, Meghana Kamineni, Winston Lie, Anoop K. Prasad, Adam Landman, Keith Dreyer, and Marc D. Succi. Assessing the utility of chatgpt throughout the entire clinical workflow: Development and usability study. *Journal of medical Internet research*, 25:e48659, August 2023.

[6] Yat-Fung Shea, Cynthia Min Yao Lee, Whitney Chin Tung Ip, Dik Wai Anderson Luk, and Stephanie Sze Wing Wong. Use of gpt-4 to analyze medical records of patients with extensive investigations and delayed diagnosis. *JAMA network open*, 6:e2325000, August 2023.

[7] Kiyoshi Shikino, Taro Shimizu, Yuki Otsuka, Masaki Tago, Hiromizu Takahashi, Takashi Watari, Yosuke Sasaki, Gemmei Iizuka, Hiroki Tamura, Koichi Nakashima, Kotaro Kunitomo, Morika Suzuki, Sayaka Aoyama, Shintaro Kosaka, Teiko Kawahigashi, Tomohiro Matsumoto, Fumina Orihara, Toru Morikawa, Toshinori Nishizawa, Yoji Hoshina, Yu Yamamoto,

Yuichiro Matsuo, Yuto Unoki, Hirofumi Kimura, Midori Tokushima, Satoshi Watanuki, Takuma Saito, Fumio Otsuka, and Yasuharu Tokuda. Evaluation of chatgpt-generated differential diagnosis for common diseases with atypical presentation: Descriptive research. *JMIR medical education*, 10:e58758, June 2024.

[8] Daisuke Horiuchi, Hiroyuki Tatekawa, Tatsushi Oura, Satoshi Oue, Shannon L. Walston, Hirotaka Takita, Shu Matsushita, Yasuhito Mitsuyama, Taro Shimono, Yukio Miki, and Daiju Ueda. Comparing the diagnostic performance of gpt-4-based chatgpt, gpt-4v-based chatgpt, and radiologists in challenging neuroradiology cases. *Clinical neuroradiology*, May 2024.

[9] Daisuke Horiuchi, Hiroyuki Tatekawa, Taro Shimono, Shannon L. Walston, Hirotaka Takita, Shu Matsushita, Tatsushi Oura, Yasuhito Mitsuyama, Yukio Miki, and Daiju Ueda. Accuracy of chatgpt generated diagnosis from patient's medical history and imaging findings in neuroradiology cases. *Neuroradiology*, 66:73–79, January 2024.

[10] Daniel Milad, Fares Antaki, Jason Milad, Andrew Farah, Thomas Khairy, David Mikhail, Charles-Édouard Giguère, Samir Touma, Allison Bernstein, Andrei-Alexandru Szigiato, Taylor Nayman, Guillaume A. Mullie, and Renaud Duval. Assessing the medical reasoning skills of gpt-4 in complex ophthalmology cases. *The British journal of ophthalmology*, February 2024.

[11] Tassallah Abdullahi, Ritambhara Singh, and Carsten Eickhoff. Learning to make rare and complex diagnoses with generative ai assistance: Qualitative study of popular large language models. *JMIR medical education*, 10:e51391, February 2024.

[12] Tomohiro Kikuchi, Takahiro Nakao, Yuta Nakamura, Shouhei Hanaoka, Harushi Mori, and Takeharu Yoshikawa. Towards improved radiological diagnostics: Investigating the utility and limitations of gpt-3.5 turbo and gpt-4 with quiz cases. *AJNR. American journal of neuroradiology*, May 2024.

[13] Alejandro Ríos-Hoyo, Naing Lin Shan, Anran Li, Alexander T. Pearson, Lajos Pusztai, and Frederick M. Howard. Evaluation of large language models as a diagnostic aid for complex medical cases. *Frontiers in medicine*, 11:1380148, 2024.

[14] Martin Krusche, Johnna Callhoff, Johannes Knitza, and Nikolas Ruffer. Diagnostic accuracy of a large language model in rheumatology: comparison of physician and chatgpt-4. *Rheumatology international*, 44:303–306, February 2024.

[15] Stephan Rau, Alexander Rau, Johanna Nattenmüller, Anna Fink, Fabian Bamberg, Marco Reisert, and Maximilian F. Russe. A retrieval-augmented chatbot based on gpt-4 provides appropriate differential diagnosis in gastrointestinal radiology: a proof of concept study. *European radiology experimental*, 8:60, May 2024.

[16] Wan Hang Keith Chiu, Wei Sum Koel Ko, William Chi Shing Cho, Sin Yu Joanne Hui, Wing Chi Lawrence Chan, and Michael D. Kuo. Evaluating the diagnostic performance of large language models on complex multimodal medical cases. *Journal of medical Internet research*, 26:e53724, May 2024.

[17] Joseph Barile, Alex Margolis, Grace Cason, Rachel Kim, Saia Kalash, Alexis Tchaconas, and Ruth Milanaik. Diagnostic accuracy of a large language model in pediatric case studies. *JAMA pediatrics*, 178:313–315, March 2024.

[18] David Li, Kartik Gupta, Mousumi Bhaduri, Paul Sathiadoss, Sahir Bhatnagar, and Jaron Chong. Comparing gpt-3.5 and gpt-4 accuracy and drift in radiology diagnosis please cases. *Radiology*, 310:e232411, January 2024.

[19] Roya Zandi, Joseph D. Fahey, Michael Drakopoulos, John M. Bryan, Siyuan Dong, Paul J. Bryar, Ann E. Bidwell, R. Chris Bowen, Jeremy A. Lavine, and Rukhsana G. Mirza. Exploring diagnostic precision and triage proficiency: A comparative study of gpt-4 and bard in addressing common ophthalmic complaints. *Bioengineering (Basel, Switzerland)*, 11, January 2024.

[20] Dik Wai Anderson Luk, Whitney Chin Tung Ip, and Yat-Fung Shea. Performance of gpt-4 and gpt-3.5 in generating accurate and comprehensive diagnoses across medical subspecialties. *Journal of the Chinese Medical Association : JCMA*, 87:259–260, March 2024.

[21] Zachary M. Tenner, Michael C. Cottone, and Martin R. Chavez. Harnessing the open access version of chatgpt for enhanced clinical opinions. *PLOS digital health*, 3:e0000355, February 2024.

[22] Thomas Savage, Ashwin Nayak, Robert Gallo, Ekanath Rangan, and Jonathan H. Chen. Diagnostic reasoning prompts reveal the potential for large language model interpretability in medicine. *NPJ digital medicine*, 7:20, January 2024.

[23] Shunsuke Koga, Nicholas B. Martin, and Dennis W. Dickson. Evaluating the performance of large language models: Chatgpt and google bard in generating differential diagnoses in clinicopathological conferences of neurodegenerative disorders. *Brain pathology (Zurich, Switzerland)*, 34:e13207, May 2024.
